# Shear-coupled grain boundary migration assisted by unusual atomic shuffling

**DOI:** 10.1038/srep23602

**Published:** 2016-03-24

**Authors:** Liang-Liang Niu, Ying Zhang, Xiaolin Shu, Fei Gao, Shuo Jin, Hong-Bo Zhou, Guang-Hong Lu

**Affiliations:** 1 Department of Physics, Beihang University, Beijing 100191, China; 2 Department of Nuclear Engineering and Radiological Science, University of Michigan, Ann Arbor, MI 48109, USA

## Abstract

Shear-coupled grain boundary (GB) migration can be an efficacious mechanism to accommodate plastic deformation when the grain size of polycrystalline materials goes small. Nevertheless, how this kind of GB motion comes into play at the atomic level has not been fully revealed. Here, we have investigated the shear-coupled migration (SCM) of typical [100] group symmetrical tilt GBs in bcc W using atomistic simulations. Depending on GB character, the SCM is found to proceed via dislocation slipping in the 〈100〉 or 〈110〉 mode with striking shear strength difference between them. We demonstrate that there exists an unusual atomic shuffling along the tilt axis, which greatly assists SCM to operate in the easier 〈110〉 mode instead of the 〈100〉 one. The present results highlight the significant role of GB character in the atomistic SCM process and contribute to the future design and fabrication of high-performance materials in GB engineering.

Shear-coupled grain boundary migration (SCM), involving the concomitant lateral translation of adjacent grains and normal grain boundary (GB) displacement, is a rather common phenomenon dating back to the 1950s when such motion was firstly found in low-angle GBs in zinc^1^. Since then, substantial experimental observations^2^,^3^,^4^,^5^,^6^,^7^,^8^,^9^ on stress-driven grain growth at low or intermediate temperature have evidenced the effectiveness of SCM among GB-mediated plasticity mechanisms in ultrafine grained or nanocrystalline metals containing both low- and high-angle GBs. Theoretically, such deformation mode was revealed to be energetically more favorable and even enhance more effectively the ductility of nanocrystalline solids than its counterpart of pure GB sliding^10^. Within the framework of coincidence site lattice and displacement shift complete lattice, Cahn *et al*.^11^,^12^ proposed a geometric model: ‘special’ GBs can be characterized by a GB geometry-dependent coupling factor, which was subsequently corroborated by a range of molecular dynamics (MD) simulations^11^,^13^,^14^,^15^,^16^ and bicrystal experiments^17^,^18^,^19^,^20^,^21^. The unified theory of Cahn has been further generalized^22^ in order to account for the *in situ* observations of SCM in general GBs.

Notably, atomistic simulations have provided considerable insights into the elementary mechanisms of SCM, which are difficult to access through experimental techniques. For example, Mishin and co-workers^14^,^15^ reported the effects of temperature and strain rate on the critical stress of SCM, showing the close analogy between SCM and other well-recognized dynamic regimes, such as stick-slip and Brownian dynamics. They^23^ further demonstrated that the coupling modes found in symmetrical tilt grain boundaries (STGBs) continue to operate in a wide range of asymmetrical tilt GBs with a coupling factor affected by more parameters. Wang *et al*.^24^ showed the significance of stacking fault energy in the sliding and migration of incoherent twin boundaries in response to shear in fcc metals. The comprehensive effects of GB character and temperature on SCM have also been uncovered^25^. Intriguingly, structural phase transitions of metallic GBs enabled by a novel simulation methodology^26^,^27^ can have huge influence on the critical stress and even the nature of SCM^16^. To date, SCM has been revealed to occur through the collective glide of GB resolved dislocations, the nucleation and motion of GB disconnections or the rotation of structural units^11^,^28^,^29^,^30^,^31^,^32^,^33^,^34^, which are all fundamentally related to the motion of GB dislocations.

Most recently, SCM has been proposed as a promising self-healing mechanism in bcc tungsten (W)^35^ and fcc nanotwinned silver (Ag)^36^. However, in spite of its scientific interest and technological importance, a complete understanding of SCM at the atomic level in metals, especially bcc ones, is still lacking. In this work, with the effects of GB character taken into account, new features regarding the microscopic mechanisms of SCM in [100] STGBs in W are revealed utilizing atomistic simulations based on MD methods. The implications of this work are discussed.

## Results and Discussion

### Grain boundary energy and structure

**Figure 1:**
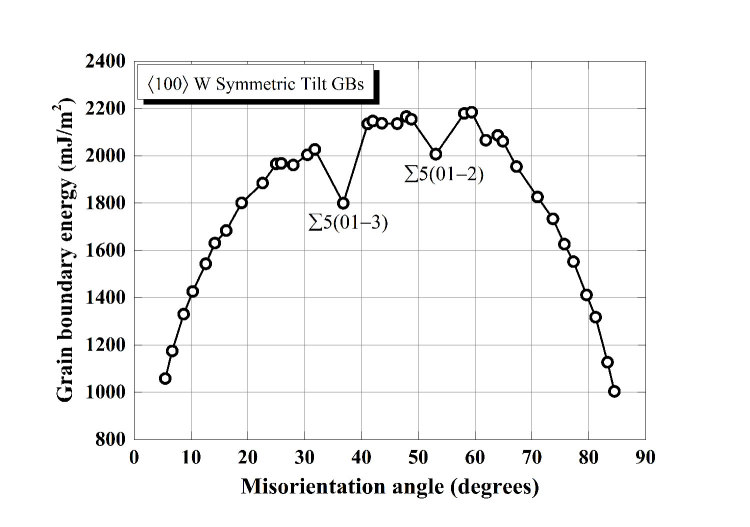
Grain boundary energy as a function of misorientation angle for the [100] STGBs in bcc W.

The energy and structure of GBs in bcc, fcc and hcp metals have been under extensive investigation for several decades^37^,^38^,^39^,^40^,^41^,^42^. Here we studied 36 [100] STGBs with misorientation angle *θ* varying between 5.5° and 84.5°. Almost all distinct GB structures have been covered within this interval for this particular GB group. Figure 1 shows the extra energies of these GBs as a function of misorientation angle, among which the GB energies of low-angle GBs (with *θ* → 0° and *θ* → 90°) are much lower than the high-angle ones. Notably, ∑5(01–3) and ∑5(01–2) GBs shown as the minor cusps stand out among the high-angle category. The results are in excellent agreement with those found in bcc Fe^37^.

**Figure 2:**
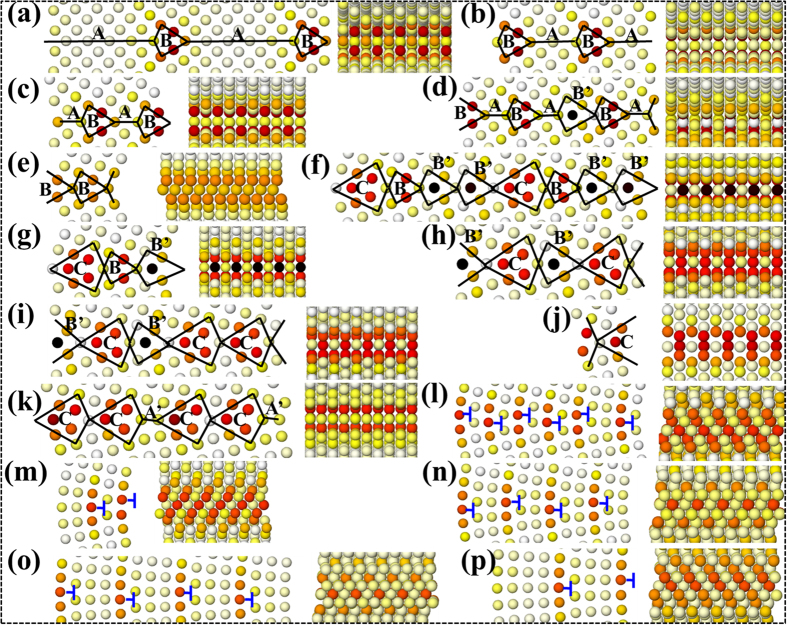
Typical equilibrium GB structures with basic structural units constituting the (**a**) ∑85(01–13) = 8.8°, (**b**) ∑25(01–7) = 16.3°, (**c**) ∑13(01–5) = 22.6°, (**d**) ∑85(02–9) = 25.1°, (**e**) ∑5(01–3) = 36.9°, (**f**) ∑97(05–13) = 42.1°, (**g**) ∑29(02–5) = 43.6°, (**h**) ∑29(03–7) = 46.4°, (**i**) ∑97(04–9) = 47.9°, (**j**) ∑5(01–2) = 53.1°, (**k**) ∑53(05–9) = 58.1°, (**l**) ∑89(05–8) = 64.0°, (**m**) ∑13(02–3) = 67.4°, (**n**) ∑37(05–7) = 71.1°, (**o**) ∑65(07–9) = 75.7° and (**p**) ∑41(04–5) = 77.3°. The left and right images are equilibrium GB structures projected onto the *yz* and *xy* planes, respectively. Atoms are colored according to their potential energies and a darker color indicates higher energy.

Typical equilibrium GB structures are illustrated in Fig. 2. At the lower end of the misorienation angle, the low-angle GBs present a discrete array of 〈100〉 dislocations with a kite-shaped core. Actually, these GBs can also be described by the type 2 tilt wall model^39^,^40^ proposed previously. At the higher end of the range, the low-angle GBs consists of an array of revolved −1/2 〈110〉 dislocations (see Fig. 1l–p), which well fit the type 1 tilt wall model^39^,^40^. As the effective misorientation angle increases, the dislocation cores approach each other and begin to overlap. The GB structures thus formed can be readily characterized by the structural unit model^43^.

Previous studies have demonstrated that GBs with noticeable energy cusps are favorable. In hcp metals, these favorable GBs correspond to coherent STGBs which serve as the base boundary structure for the STGBs neighboring them^39^,^40^; whereas in bcc and fcc metals, these favorable GBs are consisted of some basic structural units and a combination of these favored structural units forms other STGBs with complexity. As shown in Fig. 1, the ∑5(01–3) and ∑5(01–2) STGBs are clearly favored for this GB group. We have identified five basic structural units as labelled in Fig. 2. Specifically, structural units A and A’ corresponds to the (100) and (110) components of the perfect lattice, B and C are the basic units of the ∑5(01–3) and ∑5(01–2) STGBs, while B’ is a filled version of B due to the added row of atoms to minimize the system potential energy. The STGBs can be determined by a combination of structural units from the favored boundaries neighboring them. The STGBs in Fig. 1a–e are formed by the structural units of the ∑5(01–3) and that of perfect lattice, STGBs in Fig. 1f–j come from a combination of structural units of ∑5(01–3) and ∑5(01–2), while the STGB in Fig. 1k is composed of structural units of ∑5(01–2) and that of the perfect lattice. For example, ∑85(01–13) = 10A + 2B, ∑13(01–5) = 2A + 2B, ∑97(05–13) = 2B + 4B’ + 2C and ∑53(05–9) = 2A’ + 4C.

### Stick-slip dynamics of SCM

**Figure 3:**
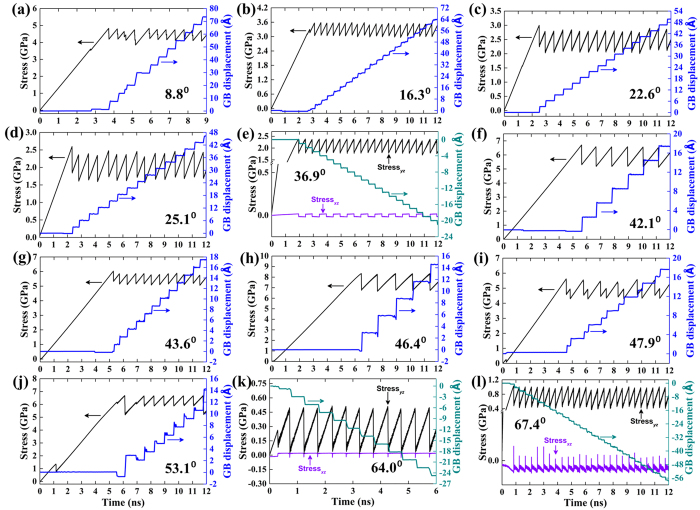
Shear stress and GB displacement as a function of time for representative STGBs of (**a**) ∑85(01–13) = 8.8°, (**b**) ∑25(01–7) = 16.3°, (**c**) ∑13(01–5) = 22.6°, (**d**) ∑85(02–9) = 25.1°, (**e**) ∑5(01–3) = 36.9°, (**f**) ∑97(05–13) = 42.1°, (**g**) ∑29(02–5) = 43.6°, (**h**) ∑29(03–7) = 46.4°, (**i**) ∑97(04–9) = 47.9°, (**j**) ∑5(01–2) = 53.1°, (**k**) ∑89(05–8) = 64.0° and (**l**) ∑13(02–3) = 67.4° at 0.1 K. The *xz* component of the shear stress is shown only in the 〈110〉 branch STGBs because it is zero for the 〈100〉 branch.

Figure 3 presents the variation of shear stress and GB displacement as a function of time at 0.1 K employing a velocity of 0.2 m/s. Note that altering the velocity by one order of magnitude has practically no influence on the elementary mechanisms of SCM, whereas the low temperature can dramatically suppress the effect of thermal noises which might jeopardize the examination of SCM. It can be seen that the *yz* component of the shear stress (*S*_*yz*_) exhibits a sawtooth behavior characteristic of stick-slip dynamics and the GB migrates in a stop-and-go manner. The stress-induced GB migration can be either positive or negative depending on GB character. According to Cahn^11^, two branches of GB geometry-dependent coupling factor *β*, characterized by a linear relation, *β = T/N,* between normal GB displacement *N* and parallel GB translation *T*, can be determined based on the slip direction of GB dislocations. The 〈100〉 branch, 
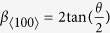
, wherein *θ* is the misorientation angle, corresponds to the positive GB migration, whereas the 〈110〉 branch, 
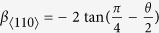
dictates the negative one.

We found that for STGBs migrating in the 〈110〉 mode, there exists an inherent stress component along *xz* (see the methods section for the crystallographic directions) in the ground state GB structure. As shown in Fig. 3e,k,l, this stress component changes periodically during the SCM process. Accompanying each step of GB displacement, *S*_*yz*_ exhibits a precipitous drop accompanied by the sign change of *S*_*xz*_ and then *S*_*xz*_ may change sign or maintain that sign until the next GB displacement. Interestingly, no noticeable *S*_*xz*_ values have been recorded in GBs moving in the 〈100〉 mode and we thus did not show them. The atomistic origins of these phenomena are illustrated in the following sections.

### Dimensionality of atomic displacement associated with SCM

**Figure 4:**
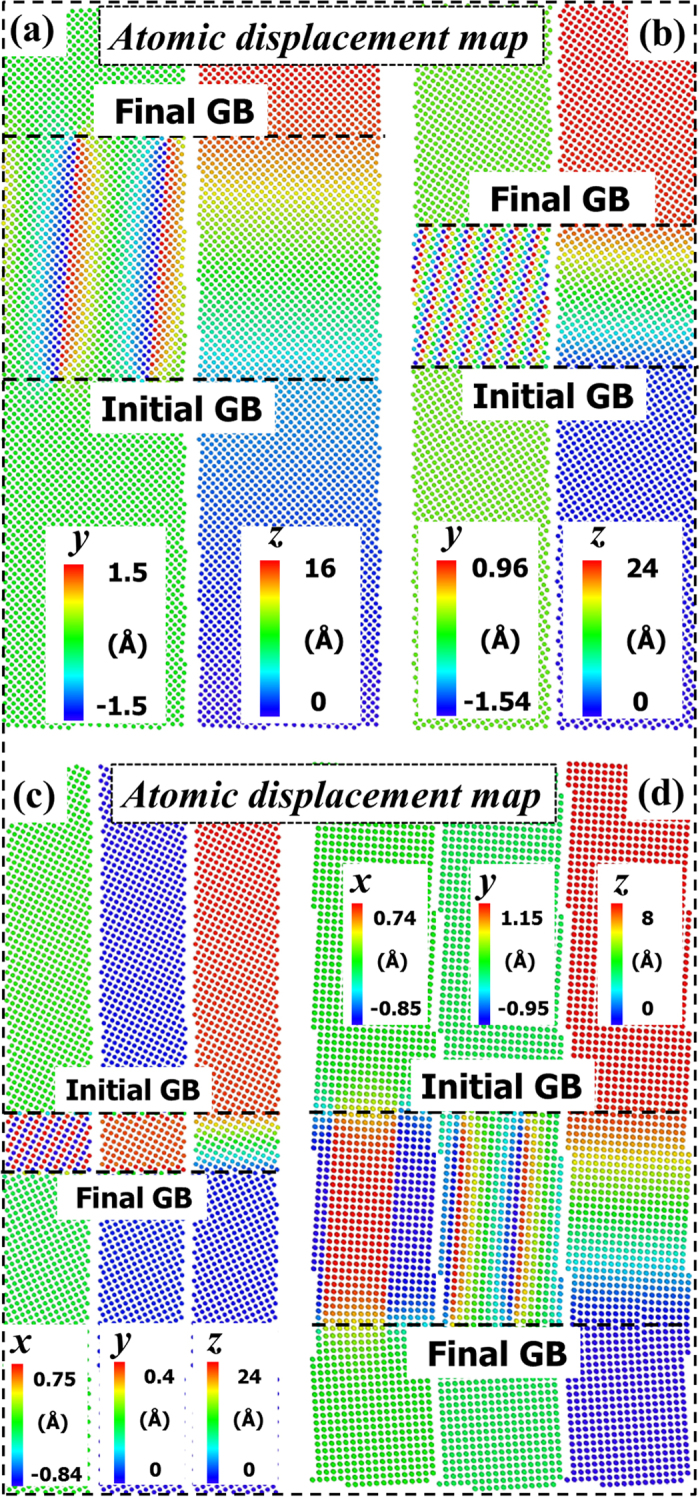
Atomic *x*-, *y*- and *z*-axial displacement maps of the final snapshot for (**a**) ∑221(01–21) = 5.5°, (**b**) ∑13(01–5) = 22.6°, (**c**) ∑5(01–3) = 36.9° and ∑145(08–9) = 83.3° at 0.1 K. The atomic *x*-axial displacement map is shown only in the 〈110〉 branch STGBs because it is zero for the 〈100〉 branch.

Next, we provide further atomic insights into the SCM regarding the dimensionality of the atomic displacements. Figure 4 shows four typical atomic displacement maps at the end of the MD simulation run, among which STGBs in (a) and (b) migrate in the 〈100〉 mode and those in (c) and (d) migrate in the 〈110〉 one. Interestingly, for STGBs moving in the 〈110〉 mode (see Fig. 4c,d), the *x*-axial displacement maps demonstrate that concomitant with each step of the SCM, atoms locating at opposite sides of the GB dislocation glide plane are found to undergo transient displacements along the tilt axis towards opposite directions. Moreover, atoms in the regions, which are both traversed by the GB migration and sandwiched between neighboring dislocation glide planes, experience the equal total *x*-axial displacements. These effects give reasonable explanations as to why *S*_*xz*_ changes sign (Fig. 3e,k,l). Note that the *y*-axial displacement maps indicate the rotation of dislocation core structures, while the *z*-axial displacement maps imply the existence of shear deformation of the region swept by the GB. In short, the atomic displacement maps demonstrate that atomic displacements in response to shear are actually two-dimensional (no *x*-axial displacements) for the 〈100〉 branch STGBs, in contrast to the three-dimensional atomic displacements of the 〈110〉 branch. The present results show that the atomic shuffling along the tilt axis in the SCM process contributes to the sign change of the shear stress component along the tilt axis, while the same shear stress component has not been observed when there is no such shuffling. The important role played by this kind of atomic shuffling will be subsequently discussed.

### SCM assisted by the tilt axis-oriented atomic shuffling

**Figure 5:**
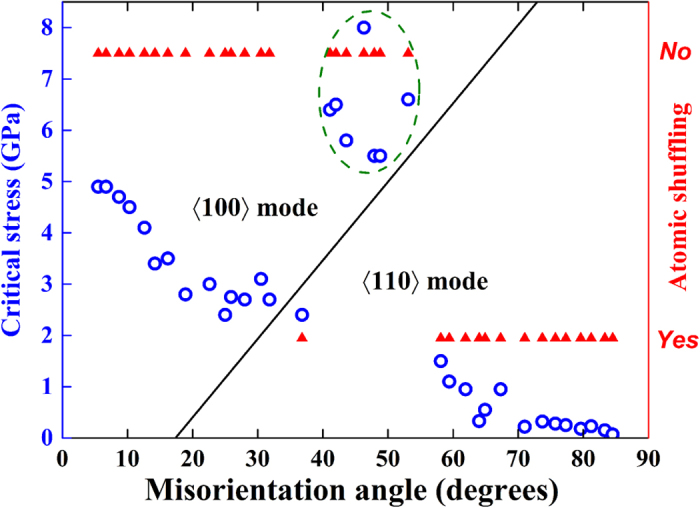
Critical stress, SCM mode and whether tilt axis-oriented atomic shuffling exists for the SCM process of the [100] group STGBs at 0.1 K. The tilt line separates the two modes of SCM. The transition from 〈100〉 mode to 〈110〉 one with increasing misorientation angle was traditionally thought to occur at *θ* ∈ (31.9°, 36.9°). The seven STGBs surrounded by the oval box that are normally expected to move in the 〈110〉 mode actually move in the 〈100〉 one due to lack of atomic shuffling along the tilt axis.

**Figure 6:**
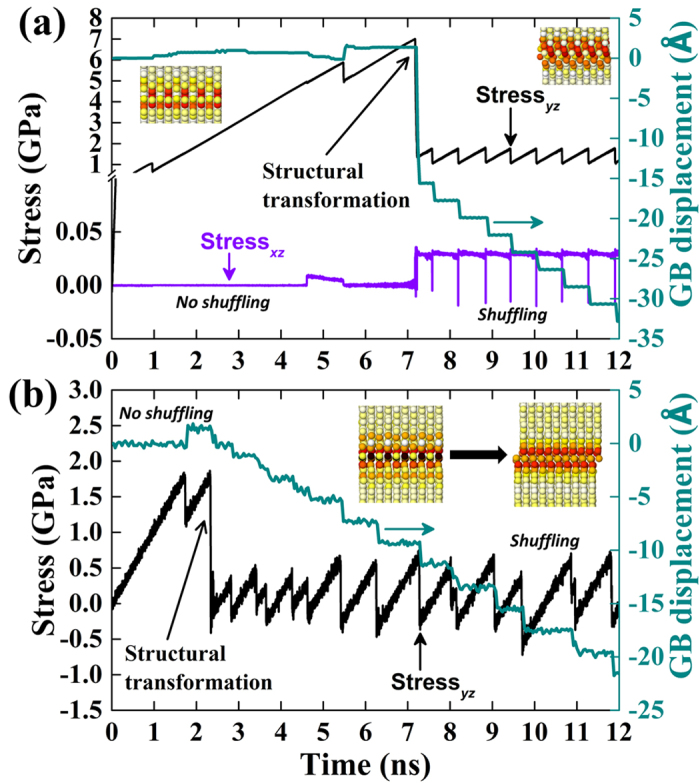
Shear stress and GB displacement as a function of time for the special case of (**a**) ∑53(05–9) = 58.1° at 0.1 K and (**b**) ∑97(05–13) = 42.1° at 300 K. The left and right inset images are the respective *xy*-plane projection of the equilibrium GB structures before and after the GB structural transformation.

In previous sections, we have pointed out that there exists an unusual tilt axis-oriented atomic shuffling associated with an intrinsic shear stress component along the tilt axis during the SCM process of the 〈110〉 branch GB. Here we try to understand how this kind of atomic shuffling correlates with the shear strength, which constitutes the central results of this work. We plot in Fig. 5 the critical stress, SCM mode, and whether tilt axis-oriented atomic shuffling exists as a function of misorientation angle. It can be seen that the critical stress for SCM generally decreases with increasing misorientation angle accompanied by mode change from 〈100〉 to 〈110〉 at *θ* ∈ (31.9°, 36.9°). Clearly, a correlation between GB energy and critical stress cannot be established. Surprisingly, seven GBs, i.e., ∑73(03–8), ∑97(05–13), ∑29(02–5), ∑29(03–7), ∑97(04–9), ∑73(05–11) and ∑5(01–2), which are supposed to move in the 〈110〉 mode according to previously defined criterion^11^,^19^, fall into the region of 〈100〉 mode with an extraordinarily high critical stress. ‘Coincidentally’, no atomic shuffling (no shear stress component along the tilt axis) can be observed for all these GBs moving in the 〈100〉 mode, whereas a relative atomic shuffling of ~*a*/2 (see Fig. 4c,d) was detected for all GBs moving in the 〈110〉 mode. In Fig. 6 and Supplementary Videos, we present further evidence to support this finding by showing the SCM of the ∑53(05–9) STGB at 0.1 K and the ∑97(05–13) STGB at 300 K. Figure 6a and Supplementary Video S1 show that no atomic shuffling was observed at the initial stage of the shearing run and the stress accumulates to a value as high as ~7 GPa. We observed slight positive GB migration in the 〈100〉 mode. Surprisingly, at ~7.2 ns, we observed pronounced structural transformation accompanied by atomic shuffling along the tilt axis and the GB quickly moves down in the 〈110〉 mode with a dramatically reduced stress level. As far as the ∑97(05–13) STGB is concerned, it moves in the 〈100〉 mode at 0.1 K in the 12 ns simulation (see Fig. 3f and Supplementary Video S2). However, once the temperature is increased to 300 K, the change of SCM mode was observed once again (see Fig. 6b and Supplementary Video S3). Specifically, at ~2.3 ns, structural transformation accompanied by atomic shuffling along the tilt axis was observed and the GB quickly moves down in the 〈110〉 mode with a greatly reduced stress level. We thus conclude that the tilt axis-oriented atomic shuffling can effectively assist the STGBs to move in a more energy-efficient 〈110〉 mode.

We further recognize that the discovered correlation between atomic shuffling, the SCM mode and critical stress can be traced back to the equilibrium GB structure (see Fig. 2). The 〈100〉 branch STGBs are highly mirror-symmetric to the GB interface, and it takes tremendous time to break the mirror symmetry at low temperatures due to the suppressed thermal fluctuations, thus they are more likely operate in the difficult 〈100〉 mode. As a matter of fact, we observed more frequent SCM mode changes from 〈100〉 to 〈110〉 induced by structural transformation with increasing temperature (e.g. Fig. 6b and Supplementary Video S3) due to the fact that high temperature increases the probability of symmetry breaking. In contrast, this kind of mirror symmetry is broken for the 〈110〉 branch STGBs at the beginning due to the shift along the tilt axis, they can, therefore, readily operate in the easier 〈110〉 mode. The present finding pinpoints the important effect of inherent GB character on the atomistic mechanism and critical stress of SCM. We remark that the atomistic mechanism revealed here is expected to be generally applicable to the SCM process of all STGBs in bcc metals and how the present mechanism depends on temperature, velocity or defects will be a subject of future work.

## Conclusions

In this work, we have conducted extensive atomistic simulations to investigate the SCM behavior of [100] STGBs in bcc W at low temperature. Analysis of the equilibrium GB structures shows that the STGBs can be adequately described by the previously proposed structural unit model and dislocation model. We show that the SCM proceeds through two modes of GB dislocation slipping ( along 〈100〉 or 〈110〉 directions) depending on GB character. The shear strength of the 〈100〉 branch GBs is much higher than the 〈110〉 ones. Surprisingly, we reveal that there exists an unusual tilt axis-oriented atomic shuffling that can effectively assist SCM to operate in the easier 〈110〉 mode. This kind of atomic shuffling is directly responsible for the sign change of the shear stress component along the tilt axis. We attribute the observed atomistic mechanism to the inherent GB equilibrium structure. Transformation of the GB structure may also simultaneously change the SCM mode. The present work points out the strong dependence of the atomic-scale SCM process on GB character. The atomistic mechanism presented here suggests another factor be taken into consideration when designing and fabricating novel GB-containing materials in GB engineering.

## Methods

**Figure 7:**
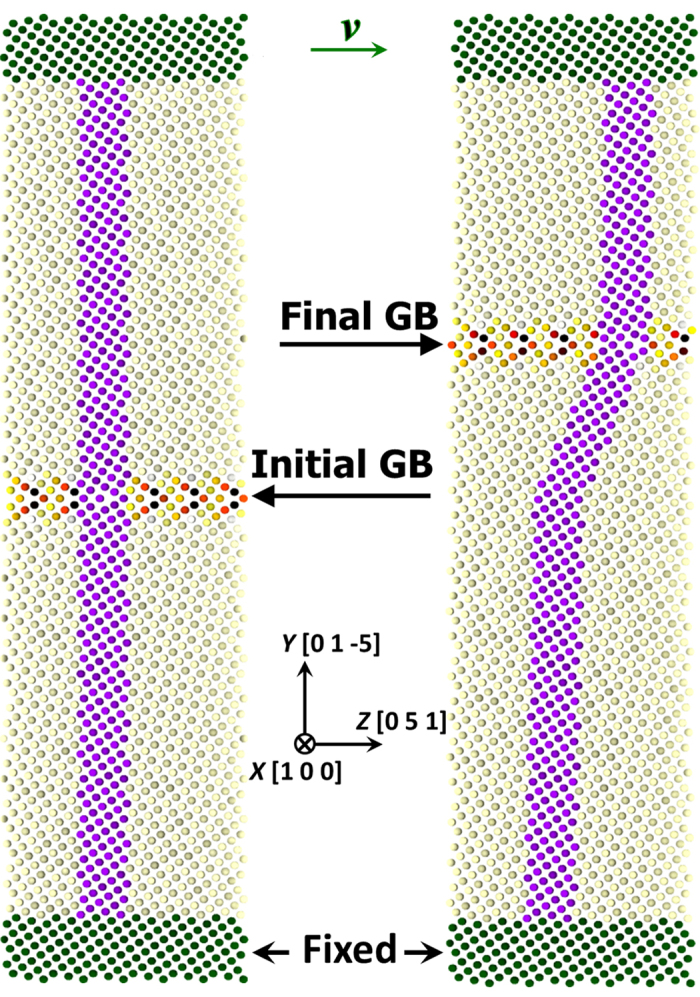
Exemplary SCM processes in the ∑13(01–5) = 22.6° STGB moving in the 〈100〉 mode. The rest STGBs follow a similar crystallographic relationship and shearing procedure. Atoms are colored according to their potential energies and a darker color indicates higher energy. The violet marker is used to portray the shear deformation.

A parallel MD package, LAMMPS^44^ is used to explore the SCM process of the [100] series STGBs constructed according to ref. 37. Firstly, based on coincident site lattice theory, two grains with different crystallographic orientations are brought together to obtain the bicrystal. Then, the upper and lower grains are translated with respect to each other in-plane by different magnitudes, followed by an atom deletion process to avoid close contact between atoms. Notably, due to the in-plane translation process, the mirror-symmetry of the STGBs is frequently observed to be broken, which has been reported in previous studies^45^,^46^. The size of the system varies depending on the specific GB type and typical bicrystal contains thousands of atoms. The interatomic potential for W-W interaction is described in previous works^47^,^48^. Periodic boundary conditions are applied parallel to the GB interface (*x* and *z* directions). For the convenience of imposing shear stress on neighbouring dynamic atoms, top and bottom slabs of ~12 Å thick (more than twice the cutoff radius) are frozen by setting the interatomic force to zero. An exemplary run of the SCM process and the crystallographic relation are illustrated in Fig. 7. A constant shearing velocity is subsequently applied to the upper slab along +*z* direction with the lower slab remaining fixed. Note that all shearing is conducted at 0.1 K with a velocity of 0.2 m/s unless specified otherwise. The position of the GB interface is tracked according to the maximum potential energy of atoms in the GB region. In addition, the standard viral stress tensor expression implemented in LAMMPS is employed to calculate the shear stress.

## Additional Information

**How to cite this article**: Niu, L.-L. *et al*. Shear-coupled grain boundary migration assisted by unusual atomic shuffling. *Sci. Rep.* **6**, 23602; doi: 10.1038/srep23602 (2016).

## Supplementary Material

Supplementary Information

Supplementary Video S1a

Supplementary Video S1b

Supplementary Video S2a

Supplementary Video S2b

Supplementary Video S3a

Supplementary Video S2b
