# Supplementary material for: Shear-coupled grain boundary migration assisted by unusual atomic shuffling
Source: Sci Rep. 2016 Mar 24;6:23602. doi: 10.1038/srep23602 (PMC4806407; doi:10.1038/srep23602)
Supplement: Supplementary Information [file srep23602-s1.docx]

**Shear-coupled grain boundary migration assisted by unusual atomic shuffling**

Liang-Liang Niu^1,2^, Ying Zhang^1^, Xiaolin Shu^1^, Fei Gao^2^, Shuo Jin^1^, Hong-Bo Zhou^1^ & Guang-Hong Lu^1^

^1^Department of Physics, Beihang University, Beijing 100191, China. ^2^Department of Nuclear Engineering and Radiological Science, University of Michigan, Ann Arbor, MI 48109, USA. Correspondence and requests for materials should be addressed to F.G. ([gaofeium@umich.edu](mailto:gaofeium@umich.edu)) and G.H.L. ([lgh@buaa.edu.cn](mailto:lgh@buaa.edu.cn)).

**Supplementary Video S1. Shear-coupled grain boundary migration (SCM) of the ∑53(05-9)= 58.1° STGB at 0.1 K projected onto the (a) *yz* and (b) *xy* planes.** The GB structural transformation occurred at ~ 7.2 ns accompanied by the SCM mode change from〈100〉to〈110〉. Atomic shuffling along the tilt axis was observed after 7.2 ns. Atoms are colored according to their potential energies and a darker color indicates higher energy.

**Supplementary Video S2. SCM of the ∑97(05-13)=42.1° STGB at 0.1 K projected onto the (a) *yz* and (b) *xy* planes.** The SCM mode is〈100〉due to the high mirror symmetry. No GB structural transformation and atomic shuffling were observed in the 12 ns video. Atoms are colored according to their potential energies and a darker color indicates higher energy.

**Supplementary Video S3. SCM of the ∑97(05-13) =42.1° STGB at 300 K projected onto the (a) *yz* and (b) *xy* planes.** The GB structural transformation occurred at ~ 2.3 ns accompanied by the SCM mode change from〈100〉to〈110〉. Atomic shuffling along the tilt axis was observed after 2.3 ns. Atoms are colored according to their potential energies and a darker color indicates higher energy.
